# Supplementary material for: The Development of a Program to Identify and Manage Apathy in Residents with Korsakoff’s Syndrome: A Qualitative Exploration of Patient, Family Caregiver, and Professional Caregiver Perspectives
Source: Geriatrics (Basel). 2025 Nov 6;10(6):146. doi: 10.3390/geriatrics10060146 (PMC12641665; doi:10.3390/geriatrics10060146)
Supplement: Supplementary file 1 [file geriatrics-3820072-supplementary.pdf › geriatrics-3820072-supplementary.pdf]

Supplementary file 1: Topic list for the interviews and focus group meeting

Person with Korsakoff's syndrome and apathy

Due to limited disease perception, questions about the person themselves were generalized to 'people with Korsakoff's syndrome' if the person responded that it did not apply to them.

Topic: recognizing apathy:

- What comes to your mind thinking of the term apathy?
- What causes apathy in your opinion?
- How do you notice apathy in yourself/ people with Korsakoff's syndrome?

Topic: Experiences in dealing with apathy:

- Who is affected by the apathy of a person with Korsakoff's syndrome?
    - How does this affect them?
- How do you deal with apathy in daily life?
- Can you explain if it's difficult/burdensome to deal with apathy or not?
- Do you think the care team has enough knowledge on apathy in people with Korsakoff's syndrome?

Topic: Pleasant activities

- What kind of activities do you find important for having a pleasant day?

Topic: Feedback on original SABA materials *(Per individual material, All materials except for Case discussion guide discussed):*

- Does the material seem fitting for you/ people with Korsakoff's syndrome?
- Is the material clear and easy to understand?

Family caregiver and Professional caregiver

Topic: Recognizing apathy

- What comes to your mind thinking of the term apathy?
- What causes apathy in your opinion?
- How do you notice apathy in the person with Korsakoff's syndrome?
- How can professional/family caregivers recognize apathy in people with Korsakoff's syndrome?
- Were you aware of the apathy before you were told by professional caregivers? *(for family caregiver only)*

Topic: Experiences in dealing with apathy:

- Who is affected by the apathy of a person with Korsakoff's syndrome?
    - How does this affect them?
- How do you deal with apathy in daily life / in your work?
- What supports you – as a family/professional caregiver- in dealing with apathy?
- What do you need - as a family/professional caregiver - to take good care of a person with dementia and apathy?
- Can you explain if it's difficult/burdensome to deal with apathy or not?
- How do you notice – as a family/professional caregiver – that you are providing a positive interaction for the person with Korsakoff's syndrome and apathy
- Do you – as a family/professional caregiver – feel you have/the care team has enough knowledge on apathy in people with Korsakoff's syndrome?
- How confident are you in your ability to manage apathy in people with Korsakoff's syndrome?

Topic: Feedback on original SABA materials *(Per individual material, all materials discussed):*

- What elements of this material are/ are not relevant for people with Korsakoff's syndrome?
- Is the material clear and easy to understand?
- Does this material add to current practice?
- How could this material best be implemented into daily practice?
